# Phospholipidase Dδ Negatively Regulates the Function of *Resistance* to *Pseudomonas syringae pv*. *Maculicola 1* (RPM1)

**DOI:** 10.3389/fpls.2018.01991

**Published:** 2019-01-18

**Authors:** Xin Yuan, Zhangying Wang, Jianzhong Huang, Hua Xuan, Zhiyong Gao

**Affiliations:** State Key Laboratory of Hybrid Rice, Key Laboratory for Research and Utilization of Heterosis in Indica Rice of Ministry of Agriculture, College of Life Sciences, Wuhan University, Wuhan, China

**Keywords:** phospholipase D, RPM1, ABA, HR, plant immunity

## Abstract

RPM1 is a plant immune receptor that specially recognizes pathogen-released effectors to activate effector-triggered immunity (ETI) in *Arabidopsis thaliana*. RPM1 triggers ETI and hypersensitive response (HR) for disease resistance. Previous reports indicated that Phospholipase D (PLD) positively regulated RPM1-mediated HR. However, single, double, and triple *pld knock-out* mutants of 12 members of the PLD family in *A. thaliana* did not show suppressed RPM1-mediated HR, indicating the functional redundancy among PLD members. In this study, we revealed that PLD could negatively regulate the function of RPM1. We found that RPM1 interacted with PLDδ, but did not interact with PLDβ1, PLDβ2, and PLDγ3. Overexpression of *PLD*δ conducted to a reduction of protein level and corresponding activity of RPM1. We found that abscisic acid (ABA) reduced the protein level of RPM1, and the ABA-induced RPM1 reduction required PLD activity and PLD-derived phosphatidic acid (PA). Our study shows that PLD plays both negative and positive roles regulating the protein level and activity of RPM1 during stress responses in plants. PLD proteins are regulating points to integrate the abiotic and biotic responses of plants.

## Introduction

Plants develop innate immunity systems to confront pathogenic invasions (Jones and Dangl, 2006). Plants have immunity receptors to recognize pathogen-associated molecular patterns (PAMPs), and stimulate PAMP triggered immunity (PTI) (Couto and Zipfel, 2016). Pathogens evolve effectors to suppress plant PTI, and plants evolve corresponding receptors to recognize effectors, and stimulate effector triggered immunity (ETI) (Cui et al., 2015). Most of the ETI receptors are characterized with a nucleotide-binding domain (NB) and a leucine-rich-repeat domain (LRR), and these NLRs stimulate strong and rapid defense responses, named hypersensitive responses (HR), and results in cell deaths at the infection sites (Baggs et al., 2017).

RPM1 is an NLR receptor in *Arabidopsis thaliana* (Grant et al., 1995). It does not directly bind its corresponding effector AvrRpm1 or AvrB, but is activated by perceiving the effector-induced phosphorylation of the guarded protein RIN4 (Mackey et al., 2002; Chung et al., 2011; Liu et al., 2011). RPM1 is a plasma-membrane associated protein, and it does not have any transmembrane domain (Boyes et al., 1998; Gao et al., 2011). RIN4, a plasma-membrane localized protein, can stabilize RPM1, and only trace amounts of RPM1 are detected in the *rin4 knockout* plant. However, the trace amounts of RPM1 are still plasma-membrane localized (Gao et al., 2011). The activation of RPM1 leads to downstream signal transductions, including the activation of phospholipase C (PLC), the influx of extracelluar Ca^2+^, the activation of phospholipase D (PLD), and the accumulation of reactive oxygen species (ROS) (Andersson et al., 2006). The calcium-channel blocker LaCl_3_ and inhibition the activity of PLC proteins and PLD proteins are able to suppress RPM1-induced HR (Grant et al., 2000; Andersson et al., 2006).

The PLD proteins are a family of enzymes that hydrolyze membrane phospholipids, such as phosphoatidyl choline (PC) and phosphoatidylethanolamine (PE), to produce phosphatidic acid (PA) and a free-head alcohol. PLD catalyzes the reaction of transferring the phosphoatidyl group to primary alcohols to form phosphatidylalcohols instead of PA (Ella et al., 1997). Thus, *n-butanol* can be used to suppress PLD-derived PA. The *A. thaliana* genome contains 12 *PLD* genes which are grouped into α, β, γ, δ, ε, and ζ six types. Each PLD has different properties in activity regulation and/or lipid preferences (Li et al., 2009). PLDs play essential roles in responses to various abiotic and biotic stresses (Bargmann and Munnik, 2006). PLDα regulates plant response on drought and salt stresses (Sang et al., 2001; Hong et al., 2008). PLDα1 mediates abscisic acid (ABA) signaling to control the stomata closure. ABA activates PLDα1 and results in the production of PA. PLDα1-derived PA binds to ABI1, a negative regulator of ABA signaling, and the binding suppresses the negative effects of ABI1 and results in the stomatal closure. Meanwhile, PLDα1 and PA interact with the Gα subunit of heterotrimeric G protein to mediate ABA inhibition of stomatal opening (Mishra et al., 2006). PLDβ1 mediates negative defense to bacterial and fungal pathogens (Zhao et al., 2013). PLDγ1 is involved in Aluminum tolerance (Zhao et al., 2011). Plasma-membrane associated PLDδ binds to microtubules and negatively regulates thermotolerance by means of microtubule disorganization (Zhang et al., 2017). PLDδ also involves in the cell wall based defense against non-host powdery mildew fungi (Pinosa et al., 2013). PLDε regulates root growth responding to Nitrogen availability (Hong et al., 2009). PLDζ involves in phosphate deficiency and salt stresses (Li et al., 2006; Ben Othman et al., 2017).

RPM1-mediated HR can be suppressed with *n-butanol*, an antagonist for PLD-derived PA, suggesting PLD and PLD-derived PA are required for HR response (Andersson et al., 2006). Based on this result, it is reasonable to hypothesize that some *pld*-*KO* mutants should display suppressed HR response. The single, double, and triple *pld-KO* mutants of the 12 PLD members were assayed for RPM1-mediated HR, and none of the mutants showed obviously deficient HR. The results were explained with the redundancy among PLD members (Johansson et al., 2014). In this study, we revealed that PLD can negatively regulate the function of RPM1. We found that RPM1 interacted with PLDδ, but did not interact with PLDβ1, PLDβ2, and PLDγ3. Overexpression of *PLD*δ obviously conducted a reduction of protein level and corresponding activity of RPM1. We found that abscisic acid (ABA) reduced the protein level of RPM1, and the ABA-induced RPM1 reduction required PLD activity and PLD-derived phosphatidic acid (PA). Thus, the PLD activity is not only a consequence of RPM1 signal transduction, but also a negative regulator for the function of RPM1.

## Materials and Methods

### Plant Growth and Mutants

*Arabidopsis thaliana* and *Nicotiana benthamiana* plants were grown in pots with autoclaved vermiculite and watered with Hoagland solution. The growth condition is at 24°C under a 16 h light/8 h dark cycle. *A. thaliana* plants for HR and disease resistance assays were grown under 8 h light/16 h dark cycle condition. All the lines of *A. thaliana* are in *Columbia-0* background. The *pldδ* mutant 12B (SALK_023247C) were obtained from ABRC. The mutant line *rpm1-3* and the transgenic line *pRPM1::RPM1-Myc/rpm1-3* (AT5) were gifts from Dr. Jeff. Dangl (University of North Carolina at Chapel Hill, Chapel Hill, NC, United States).

### Vector Construction

The gateway system was used to construct vectors. For transient expression, The CDSs of *AtPLD* genes were cloned into the expression vector pEarleyGate 101 containing the constitutive high-expression CaMV 35S promoter, and YFP-HA tag (Karimi et al., 2007). HA tag was used for protein detection. *RPM1-Myc* was cloned into the pGWB2 vector to obtain the *35S::RPM1-Myc* expression construct (Nakagawa et al., 2007). *RPM1(D505V)-Myc* was cloned into the pMDC7 vector under the control of the estradiol-inducible promoter to obtain the *Est:: RPM1(D505V)-Myc* expression construct (Karimi et al., 2007). For the bimolecular fluorescence complementation (BiFC) assay, we modified the pEarleyGate 101 vector into vectors that could express the two complementary parts of YFP, the N terminus (nYFP) and the C terminus (cYFP) (Walter et al., 2004; Citovsky et al., 2006). The expression constructs of *35S:: RPM1 -nYFP-HA* and *35S:: PLDδ-cYFP-HA* were made respectively.

### Transient Expression in the Leaves of *N. benthamiana*

The expression constructs were electro-transformed into *Agrobacterium tumefaciens* GV3101. *Agrobacteria* were cultured overnight at 28°C with suitable antibiotics. Overnight-grown *Agrobacteria* were centrifuged and re-suspended in the induction buffer (10 mM MES pH 5.6, 10 mM MgCl_2_, 150 μM acetosyringone). Samples were infiltrated into the leaves of 5-week-old *N. benthamiana* with a 1-mL needless syringe at desired OD_600__nm_ values. *Agrobacteria* containing the P19 plasmid was always co-infiltrated with the samples at an OD_600__nm_ of 0.2. The transiently expressed proteins were extracted and analyzed at 48 h after infiltration (Pitino et al., 2016).

### Protein Extraction and Co-immunoprecipitation (Co-IP) Assay

For protein expression, three leaf disks (7 mm diameter) were ground with 110 μL of the protein extraction buffer (20 mM Tris-HCl PH 8.0, 5 mM EDTA, 10 mM DTT, 1% SDS). The supernatants were collected with centrifugation at 8,000 × *g* for 3 min and mixed with fourfold protein loading buffer. Actin was used as an internal protein loading control. For the fractioning experiments, leaf tissues were ground in a mortar with the extraction buffer (20 mM Tris-HCl pH 8.0, 150 mM NaCl, 20% glycerol, 1 mM DTT, 10 mM EDTA, 1 mM PMSF, protease inhibitor cocktail). Samples were centrifuged at 6,000 × *g* for 10 min at 4°C to remove the debris. The supernatant containing total proteins (T) was separated into the cytosolic fraction (S) and the microsomal fraction (M) with centrifugation at 20,000 × *g* for 60 min at 4°C. The plasma-membrane localized H^+^-ATPase was used as the microsomal marker. The big subunit of Rubisco was stained with Ponceau S as the cytosolic marker.

For Co-IP experiment, because PLDδ and RPM1 are membrane associated proteins, the microsomal fractions were used for Co-IP. The protein content of RPM1-Myc in the microsomal fractions were determined with Western blots, and the microsomal fractions were re-suspended with the suspension buffer (40 mM Tris-HCl PH 7.5, 150 mM NaCl, 1 mM DTT, 5 mM EDTA, protease inhibitor cocktail, 1% Triton X-100) to equalize RPM1-Myc protein levels between the control and test samples. The samples were rotated at 4°C for 1 h and centrifuged at 20,000 × *g* for 1 h to remove the insoluble proteins. 30 μL of the anti-GFP conjugated agarose beads (MBL, #D153-8) were added into 1 mL of the supernatants and incubated at 4°C for 3 h. Beads were washed three times with the suspension buffer. Immunoprecipitates were eluted with 50 μL of the elution buffer (2% SDS) at 37°C for 5 min.

The primary antibodies used for Western blots were the anti-Myc (GeneScript, #A00704), the anti-HA (Roche, #11867423001), the anti-H^+^-ATPase (Agrisera, #AS07260-100), the anti-*β*-Actin (Abbkine, #A01050-2), and the anti-T7 (Novagen, #69522).

### Confocal Microscopy

The nYFP-tagged proteins and the cYFP-tagged proteins were co-expressed in the leaves of *N. benthamiana*. The fluorescent images were observed using confocal microscopy according to the BiFC protocol (Walter et al., 2004). YFP was excited at 488 nm and fluorescent emissions were observed at 518–540 nm.

### Disease Resistance and Ion-Leakage Assay

*Pseudomonas syringae* pv. *tomato* strain DC3000(*avrB*) was grown on King’s B medium with antibiotics at 28°C for 36 h. For spray inoculation, leaves from 5-week-old plants were sprayed with *Pto* DC3000(*avrB*) in 10 mM MgCl_2_ with 0.025% silwet L-77 at an OD_600_ of 0.05. Inoculated plants were covered with a plastic dome for 2 days. All experiments were repeated three times. For ion-leakage assay, five leaf disks (8 mm diameter) of *A. thaliana* or *N. benthamiana* were floated in a clean wild-mouth tube with 6 ml of distilled water. The conductivity of the samples was measured at the indicated time points with a conductivity meter (Hubert et al., 2009).

## Results

### RPM1 Interacts With PLDδ *in planta*

**FIGURE 1:**
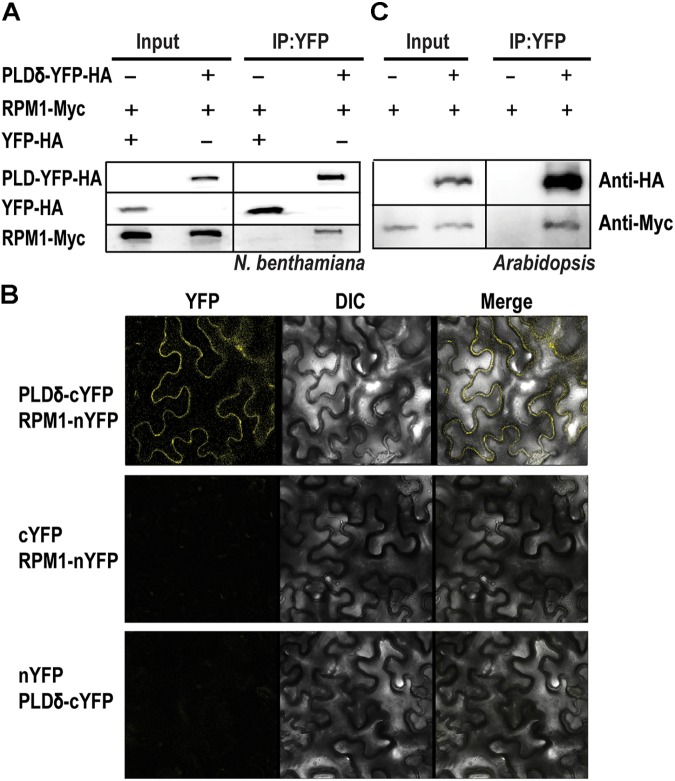
PLDδ interacts with RPM1. **(A)** The interaction of PLDδ and RPM1 in *Nicotiana benthamiana*. *35S::PLDδ-YFP-HA* and *35S::RPM1-Myc* were co-expressed in the leaves of *N. benthamiana*. Total membrane proteins were extracted from samples collected at 2 days after infiltration. PLDδ-YFP-HA was immunoprecipitated with agarose beads conjugated with anti-GFP antibody, and RPM1-Myc was detected for Co-IP. Co-expression of *35S::YFP-HA* and *35S::RPM1-Myc* was used as the negative control. **(B)** Representative confocal images of BiFC. BiFC analysis was performed in *N. benthamiana*. PLDδ was fused with the C-terminal portion of YFP (cYFP), and RPM1 was fused to the N-terminal portion of YFP (nYFP). Different plasmids were co-expressed in *N. benthamiana*. Images were pictured at 2.5 days after infiltration. All the experiments were repeated three times with similar results. **(C)** The interaction of PLDδ and RPM1 in *Arabidopsis thaliana*. Co-IP assay was performed with stable transgenic plants containing *35S::PLDδ-YFP-HA* and *pRPM1::RPM1-Myc*. The transgenic plants containing *pRPM1::RPM1-Myc* was used as the negative control.

Phospholipase D mediates the signal transduction of RPM1 (Andersson et al., 2006). We cloned and transiently expressed all the members of *AtPLD* family in the leaves of *N. benthamiana* to determine their interactions with RPM1. Of the 12 PLD members, PLDδ, PLDα2, PLDβ1, PLDβ2, and PLDγ3 had detectable protein expression, and were used for Co-IP assay. We co-expressed *35S::PLD-YFP-HA* and *35S::RPM1-Myc* in the leaves of *N. benthamiana*, and immunoprecipitated PLD-YFP-HA with the anti-GFP antibody to determine the Co-IP of RPM1-Myc. Co-expression of *35S::YFP-HA* and *35S::RPM1-Myc* was used as the negative control. The Co-IP assay showed that PLDα2 and PLDδ interacted with RPM1, while PLDβ1, PLDβ2, and PLDγ3 did not (Table 1). PLDδ was used for subsequent experiments due to stronger interaction with RPM1 (Figure 1A). We validated the interaction with the BiFC assay. The Yellow fluorescence protein (YFP) was split into two complementary parts, the N terminus (nYFP) and the C terminus (cYFP) (Walter et al., 2004; Citovsky et al., 2006). The expression constructs *35S:: RPM-nYFP-HA* and *35S:: PLDδ -cYFP-HA* were co-expressed in the leaves of *N. benthamiana*. The results showed clear yellow fluorescence in the samples co-expressing RPM1-nYFP-HA and PLDδ-cYFP-HA, but no fluorescence was detected in the negative controls (Figure 1B). We transformed *35S::PLD*δ*-YFP-HA* into the *pRPM1::RPM1-Myc/rpm1-3* plant (AT5) to determine the interaction of RPM1 and PLDδ in *A. thaliana*. The Co-IP of RPM1-Myc with PLDδ-YFP-HA in the transgenic plants indicated the interaction of RPM1 and PLDδ in *A. thaliana* (Figure 1C).

**Table 1:** Phospholipase D (PLD) genes and their protein expressions in *N. benthamiana.*

PLD genes	Detectable expression in *N. benthamiana*	Detectable interaction with RPM1
PLDδ (AT4G35790)	Yes	Yes
PLDα2 (AT1G52570)	Yes	Yes
PLDβ1 (AT2G42010)	Yes	No
PLDβ2 (AT4G00240)	Yes	No
PLDγ3 (AT4G11840)	Yes	No
PLDα1 (AT3G15730)	No	N/A
PLDα3 (AT5G25370)	No	N/A
PLDε (AT1G55180)	No	N/A
PLDζ1 (AT3G16785)	No	N/A
PLDζ2 (AT3G05630)	No	N/A
PLDγ1 (AT4G11850)	No	N/A
PLDγ2 (AT4G11830)	No	N/A


### PLDδ Suppresses RPM1(D505V)-Induced Cell Death in *N. benthamiana*

**FIGURE 2:**
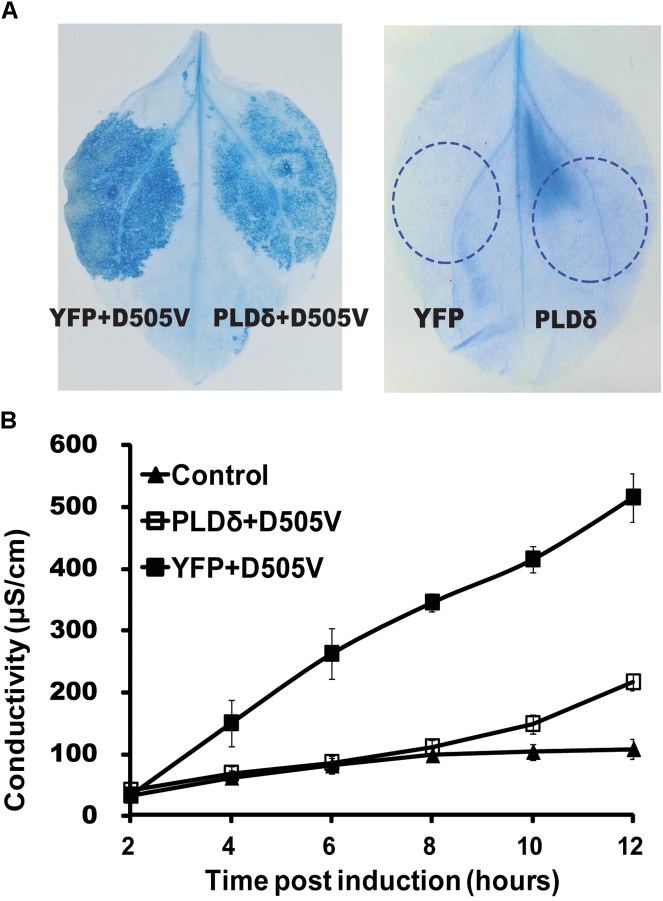
PLDδ suppresses RPM1(D505V) induced cell death in *N. benthamiana*. **(A)** Trypan blue staining of the infiltrated leaves of *N. benthamiana*. *35S::PLDδ-YFP-HA* or *35S::YFP-HA* were co-expressed with *Est::RPM1(D505V*)*-Myc*. RPM1(D505V) was induced with 20 μM estradiol at 2 days after infiltration and the leaves were stained at 6 h after induction. Leaves transiently expressed *35S::PLDδ-YFP-HA* or *35S::YFP-HA* were stained at 2 days after infiltration. **(B)** Ion-leakage assay. Leaf disks were collected from estradiol induced leaves and floated in distilled water. The conductivity of the water was measured at indicated time points. Each assay was performed with three duplicates. The error bars represent 2X standard deviation (SD). Leaves transiently expressed *35S::YFP-HA* were used as control.

Since PLDδ interacted with RPM1, we tested whether PLDδ affected the function of RPM1. We firstly determined whether PLDδ could affect RPM1-induced HR. RPM1(D505V), which mimics the active state of RPM1, is sufficient to induce the cell death (Gao et al., 2011). The estradiol inducible *Est::RPM1(D505V)-Myc* construct was co-expressed with *35S::PLDδ-YFP-HA* or *35S::YFP-HA* in the leaves of *N. benthamiana* to compare the occurrences of the cell death induced by RPM1(D505V). The cell death could be stained with trypan blue or be quantified with ion-leakage assay. Results showed that PLDδ-YFP-HA could obviously suppress RPM1(D505V) induced cell death (Figures 2A,B). We hypothesized that PLDδ either suppressed the activation of RPM1, or reduced the protein level of RPM1 to affect its function.

### PLDδ Reduces the Protein Level of RPM1(D505V) in *N. benthamiana*

**FIGURE 3:**
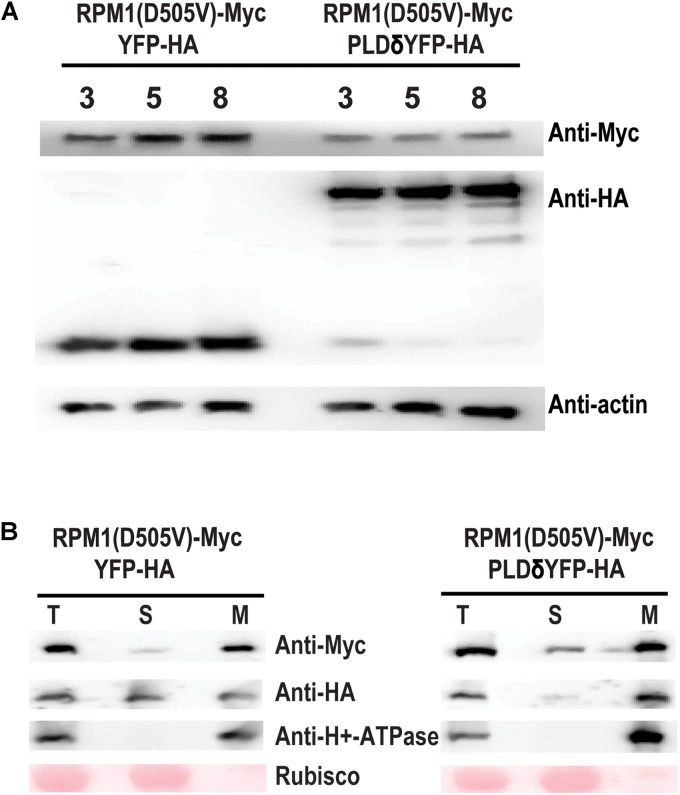
PLDδ reduces the protein level of RPM1(D505V) in *N. benthamiana*. **(A)** Protein level of RPM1(D505V). *Est::RPM1(D505V)-Myc* and *35S::PLDδ-YFP-HA* were co-expressed in the leaves of *N. benthamiana*. RPM1(D505V) was induced with estradiol at 2 days after infiltration and the protein levels of RPM1(D505V)-Myc were detected at 3, 5, and 8 h after induction. 4 mM of LaCl_3_ was infiltrated into the leaves at 2 h before induction to block RPM1(D505V) induced cell death. Co-expression of YFP-HA and RPM1(D505V) was used as a control. Actin was used as a marker for protein equal loading. **(B)** PLDδ affects the membrane association of RPM1(D505V) in *N. benthamiana.* Samples were collected at 5 h after induction. The protein extraction solution (T) was separated into the cytosolic fraction (S) and the microsomal fraction (M) using centrifugation. The distribution of RPM1(D505V)-Myc in the two fractions were detected. The large subunit of Rubisco was used as a cytosolic protein marker. The plasma-membrane protein H^+^-ATPase was used as a microsomal protein marker. The RPM1(D505V)-Myc images were adjusted to the similar density to compare RPM1(D505V) levels distributed in the S fraction. The original western blots are available in Supplementary Materials.

We tested whether PLDδ could affect the protein level of RPM1(D505V) in the leaves of *N. benthamiana*. RPM1(D505V) is unstable due to the cell death induced by the protein, and it is hard to accumulate detectable protein level. Lanthanum chloride (LaCl_3_) can inhibit RPM-mediated HR, and prevent the degradation of the activated RPM1 or RPM1(D505V) (Grant et al., 2000; El Kasmi et al., 2017). We co-expressed *Est::RPM1(D505V)-Myc* and *35S::PLDδ-YFP-HA* or *35S::YFP-HA* in the leaves of *N. benthamiana*, and induced the expression of RPM1(D505V) with estradiol at 2 days after infiltration. The leaves were treated with 4 mM of LaCl_3_ at the same time of the induction. The protein levels of RPM1(D50V) were detected at 3, 5, and 8 h after induction. Our results showed that PLDδ-YFP-HA obviously reduced the protein level of RPM1(D505V) compared with the negative control YFP-HA (Figure 3A).

RPM1(D505V) is a plasma-membrane associated protein. We collected the samples at 5 h after induction, and separated total proteins into the soluble and the microsomal membrane fractions using ultracentrifugation. Certain amount of RPM1(D505V) participated in the soluble fraction of the sample co-expressed with PLDδ, while less amount of RPM1(D505V) participated in the soluble fraction of the sample co-expressed with the negative control YFP-HA (Figure 3B).

### PLDδ Activity Is Required to Negatively Regulate the Function of RPM1(D505V) in *N. benthamiana*

**FIGURE 4:**
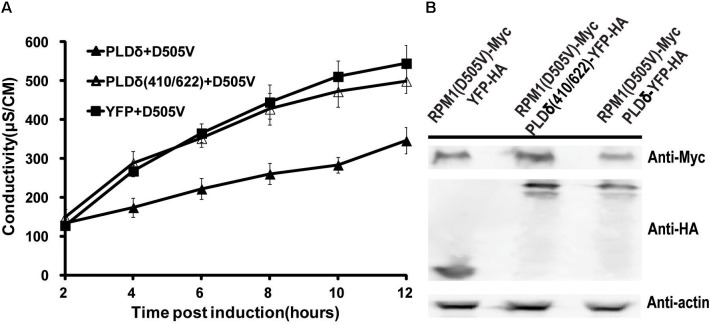
PLDδ activity is required to negatively regulate the function and the protein level of RPM1(D505V) in *N. benthamiana*. **(A)** PLDδ(R410D/R622P) is deficient to suppress the autoactivity of RPM1(D505V). *Est::RPM1(D505V)-Myc* was co-expressed with *35S::PLDδ-YFP-HA, 35S::PLDδ(R410D/R622P)-YFP-HA*, or *35S::YFP-HA* in the leaves of *N. benthamiana*. RPM1(D505V) was induced with estradiol at 2 days after infiltration. The autoactivity of RPM1(D505V) in the samples was measured with ion-leakage. **(B)** PLDδ(R410D/R622P) is deficient to reduce the protein level of RPM1(D505V). Samples were collected at 5 h after induction. The protein levels of RPM1(D505V)-Myc, PLDδ(R410D/R622P)-YFP-HA, and PLDδ-YFP-HA were detected. Actin was used as a marker for protein equal loading.

PLDδ is an oleate and phosphatidylinositol 4,5-bisphosphate (PIP2) activated enzyme. PLDδ(R410D) and PLDδ(R622P) are two activity-deficient mutants due to loss oleate binding or PIP2 stimulated activity respectively (Wang and Wang, 2001). We made PLDδ(R410D/R622P) double mutant to determine whether PLD activity was required to affect the function of RPM1(D505V). Contrast to PLDδ, the activity deficient mutant did not affect the autoactivity and the protein level of RPM1(D505V) (Figures 4A,B).

### Overexpression of PLDδ Negatively Affects the Function of RPM1 in *A. thaliana*

**FIGURE 5:**
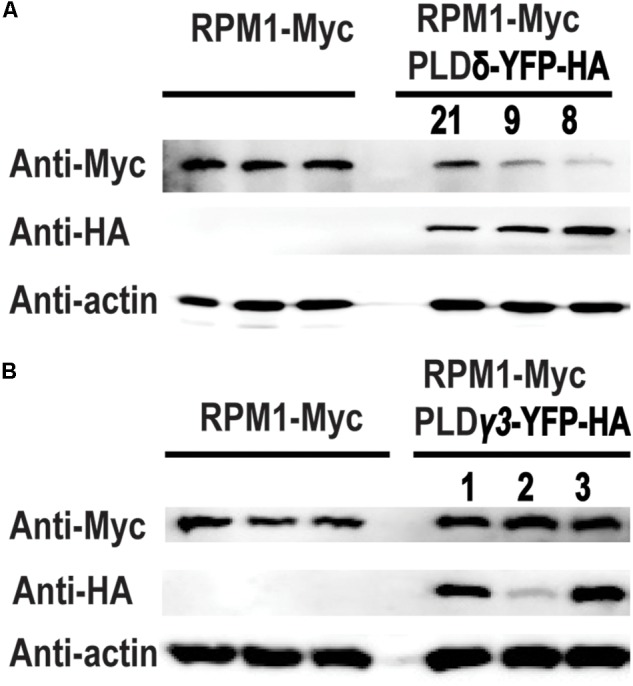
Overexpression of *PLDδ* affects the protein level of RPM1 in *A. thaliana*. **(A)** The protein level of RPM1 in the *PLDδ-OE* plants. Protein samples were collected from 5-week-old plants. Protein levels of RPM1-Myc in three independent *PLDδ OE* transgenic plants (PLD8, PLD9, and PLD21) and their background plant *pRPM1::RPM1-Myc*/*rpm1* were detected. **(B)** The protein level of RPM1 in the *PLDγ3-OE* plants. The original western blots are available in Supplementary Materials.

We transformed *35S::PLDδ-YFP-HA* into *AT5* plant to determine the effects of PLDδ on RPM1 in *A. thaliana*. Three independent transgenic lines *PLD-8, PLD-9*, and *PLD-21* were used to assay the protein level of RPM1. The results showed that the protein levels of RPM1 were lower in the *PLDδ* transgenic plants (Figure 5A). According to our assay, PLDγ3 did not interact with RPM1 in *N. benthamiana*. We transformed *35S::PLDγ3-YFP-HA* into *AT5* plant to determine the protein levels of RPM1 in three independent *PLDγ3-OE* plants. The results showed that the protein levels of RPM1 in the transgenic plants were the same as that in *AT5* (Figure 5B). The results suggested that the interaction of PLD with RPM1 was necessary to reduce the protein level of RPM1.

**FIGURE 6:**
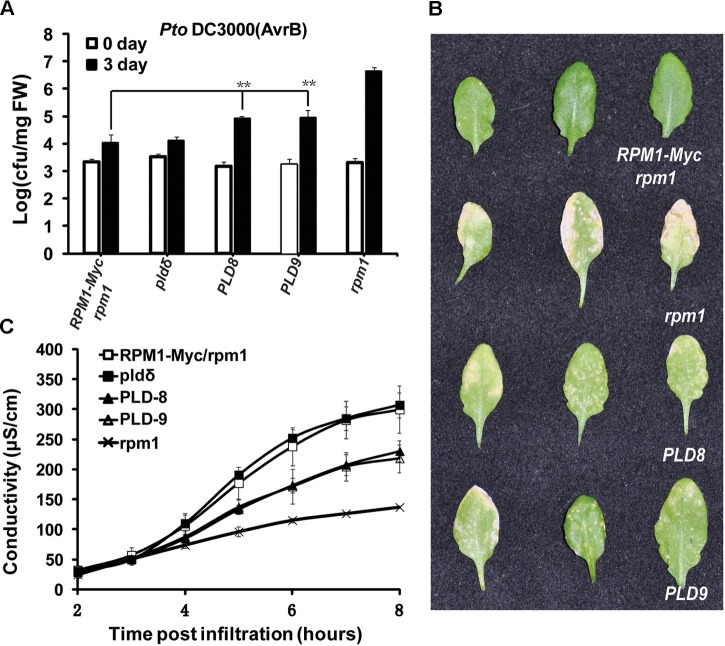
Overexpression of PLDδ suppresses RPM1-mediated disease resistance in *A. thaliana.* **(A)** Bacteria growth assay. *Pto* DC3000(*avrB*) was spray-inoculated to plant leaves at an OD_600_ of 0.05. Bacteria numbers were measured at 0 and 3 days after inoculation. The assay was performed with four duplicates. Error bars represent 2X SD. The significant difference (*P* < 0.01) is marked as ^∗∗^. PLD8 and PLD9 are two independent *PLDδ-OE* transgenic plants. **(B)** Symptoms of the inoculated leaves. Pictures were taken at 5 days after inoculation. **(C)** Ion-leakage assay. DC3000(*avrB*) was infiltrated into plant leaves at an OD_600_ of 0.05. Leaf disks were collected at 1 h after infiltration.

Since the protein level of RPM1 reduced in the *PLDδ-OE* plants, we reasoned that the function of RPM1 should be deficient in the *PLDδ-OE* plants. We compared the functions of RPM1 in *PLD-8* and *PLD-9* with those in *AT5* plant. The loss-of-function mutant *rpm1-3* was used as a negative control, and the *pldδ-KO* mutant (SALK_023247C) was included for the assays. RPM1 can be activated by its corresponding effector AvrB, and thus restrict the growth of the avirulent pathogen *Pto* DC3000(*avrB*). We spray-inoculated the leaves of the plants with *Pto* DC3000(*avrB*), and counted bacteria numbers at 0 and 3 days after inoculation. The result indicated that *PLD-8* and *PLD-9* plants displayed obviously less disease resistance than *AT5* (Figures 6A,B). The *pldδ-KO* mutant displayed the same bacteria growth restriction as *AT5*, which was consistent with previous report. We quantified the RPM1-mediated HR by measuring the ion-leakage of the plants. The ion-leakage data showed that the *PLD-8* and *PLD-9* had weaker HR than *AT5* (Figure 6C). Based on above results, we concluded that overexpressing of PLDδ reduced the protein level of RPM1, and thus negatively affected the function of RPM1.

### ABA-Induced PLD Activity Reduces the Protein Level of RPM1

**FIGURE 7:**
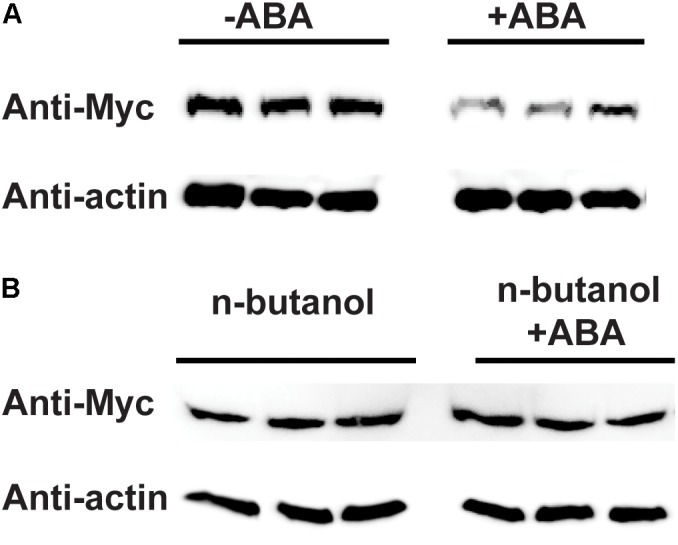
Abscisic acid (ABA)-induced PLD activity negatively regulates the protein level of RPM1. **(A)** ABA treatment reduces the protein level of RPM1. The leaves of *pRPM1::RPM1-Myc*/*rpm1*plants were incubated with or without 100 μM of ABA. Protein samples were collected at 3 h after ABA treatment. Three duplicates were detected. **(B)** *n-butanol* suppresses ABA-induced RPM1reduction. Plant leaves were incubated with 100 μM of ABA and 1% *n-butanol*. The leaves were incubated with 1% *n-butanol* were used as the negative control. The protein levels of RPM1-Myc were compared between the two treatments. Three duplicates were detected. The original western blots are available in Supplementary Materials.

Because PLD is enzyme, we want to determine whether PLD activity is required to affect the protein level of RPM1. ABA was able to stimulate the activity of PLD (Jacob et al., 1999), so we treated the leaves of *AT5* with or without 100 μM of ABA, and compared the protein levels of RPM1 between the samples. The protein levels of RPM1 assayed at 3 h after treatments, and the results showed that ABA treatment obviously reduced the protein level of RPM1 (Figure 7A). Since PLD activity leads to the synthesis of PA, we treated the plants with *n-butanol* to block the synthesis of PA and thus the PLD enzymatic function. When the plants were treated with both *n-butanol* and ABA at the same time, ABA-induced RPM1 reduction was abolished (Figure 7B). The results indicated that PLD activity and its derived PA were required to mediate the ABA-induced RPM1 reduction.

## Discussion

We revealed that PLD could negatively regulate the function of RPM1 (Figure 6), but this did not conflict with previous finding that PLD positively regulated RPM1-mediated HR. As an immune receptor, RPM1 has pro-signaling inactive state and active state. RPM1 is activated by its cognate effectors. We detected the protein levels of RPM1 in *PLDδ-OE* plants and ABA-treated plants without the inoculation of pathogenic bacteria. The results reflect the protein levels of the inactive RPM1. Since the gene expression of *RPM1* is not induced by pathogens, the protein level of the inactive pro-signaling RPM1 determines the output of the RPM1-initiated signaling (Adams-Phillips et al., 2008). PLD negatively regulates the function of RPM1 by reducing the protein level of the pro-signaling inactive RPM1. Previous report indicated that PLD played a positive role on the function of RPM1 (Andersson et al., 2006). PLD activity and its derived PA are downstream components in the RPM1 signal transduction pathway, and their positive roles are effective after the activation of RPM1. PLD activity induced by RPM1 activation may also negatively regulate the protein level and function of RPM1, but active RPM1 is quickly degraded accompanying with the HR response (Boyes et al., 1998), indicating the potential negative regulation of active RPM1 by PLD activity is not necessary.

The exact mechanism on how PLD reduces the protein level of RPM1 is not clear. Overexpression of PLDδ but not PLDγ3, which did not interact with RPM1, reduced the protein level of RPM1 in *A. thaliana*, suggesting the PLD-RPM1 interaction is required (Figure 5). Our study indicates that PLD activity is also required to reduce RPM1 level. We found that the protein level of RPM1 obviously reduced at 3 hours after ABA treatment, and PLD activity is required for ABA-induced RPM1 reduction in *Arabidopsis* (Figure 7). In addition, PLDδ(R410D/R622P), an activity-deficient mutant, could not reduce the protein level of RPM1(D505V) in *N. benthamiana* further demonstrated the necessity of PLD activity to reduce the protein level of RPM1 (Figure 4).

Transiently expressed PLD resulted in the partial distribution of RPM1(D505V) to the soluble fraction of plant cells, suggesting that PLD could interfere the plasma-membrane location of RPM1 (Figure 3B). RPM1 does not have transmembrane domains. The isoelectric point of RPM1 is 8.51, and it should be positively charged at physiological conditions. The potential interaction of the positively charged RPM1 with the negatively charged phospholipids may facilitate the membrane location of RPM1. PLD can hydrolyze PC and PE to PA which still is negative charged. It seemed that PLD activity would not affect RPM1 location. However, PA can be further dephosphated by phosphatidic acid phosphohydrolase (PAP) to diacylglycerol (DAG) (Becker and Hannun, 2005). Therefore, PLD activity should result in the net increase of DAG and decrease of PC and PE. Because DAG does not contain negatively charged phosphate group, RPM1 is released from the membrane and degraded in the cytosol of the cells. It would be interested to determine the binding of RPM1 with phospholipids such as PE and PC, and compare the lipid components between *PLDδ OE* plants and the wild type or plants treated with and without ABA treatment (Wang and Wang, 2001; Andersson et al., 2006). PLDδ reduced the protein level of RPM1(D505V), a mimic mutant of the active RPM1, in *N. benthamiana*, but PLDδ did not obviously reduce the protein level of RPM1 in *N. benthamiana*. The different effects of PLDδ on RPM1(D505V) and RPM1 reflect the conclusion that PLDδ activity is required to reduce the protein level of RPM1. RPM1-Myc itself is not active in *N. benthamiana*. Overexpression of *PLDδ* does not definitely lead to PLD activity, especially the transiently expressed PLDδ only accumulated two days. RPM1(D505V) is autoactive, and can stimulate PLD activity. Therefore, PLDδ obviously reduces the protein level of RPM1(D505V) in *N. benthamiana.* The reason that overexpression of *PLDδ* reduced the protein level of RPM1 in *Arabidopsis* (Figure 5A) is possibly due to the long-term effects of PLDδ during the time its activity can be stimulated by environmental and physiological factors (Li et al., 2009).

PLD mediates the cross-talking between ABA signaling and RPM1-mediated disease resistance. We found that ABA treatment could reduce the protein level of RPM1 in *A. thaliana.* ABA is a phytohormone responding to the drought stress, while RPM1 is an immune receptor for disease resistance. ABA signaling activates PLD, and PLD activity regulates the protein level of RPM1 and its function. Therefore, PLD could be an integrating point to balance the plant responses to the complex environmental stimuli. Other abiotic stresses such as cold, heat, and ROS can also affect PLD activity (Li et al., 2009; Guo et al., 2012; Zhang et al., 2017). It has been reported that temperature affects RPM1 function (Wang et al., 2009). PLD activity may mediate this correlation. Although we clearly determined that PLDδ could negatively regulate the protein level of RPM1, we haven’t tested all the members of the PLD family yet. Since each PLD member has specific and overlapping functions, further research about the effects of PLD members with RPM1 and other immune receptors will help to understand how PLD proteins integrate environmental and physiological responses with disease resistances.

## Author Contributions

XY and ZG designed the experiments, analyzed the data, and wrote the manuscript. XY, ZW, JH, and HX conducted the experiments.

## Conflict of Interest Statement

The authors declare that the research was conducted in the absence of any commercial or financial relationships that could be construed as a potential conflict of interest.
