# Supplementary material for: Phospholipidase Dδ Negatively Regulates the Function of Resistance to Pseudomonas syringae pv. Maculicola 1 (RPM1)
Source: Front Plant Sci. 2019 Jan 18;9:1991. doi: 10.3389/fpls.2018.01991 (PMC6345720; doi:10.3389/fpls.2018.01991)
Supplement: Supplementary file 1 [file Image_1.pdf]

Explanation on Figure 3A

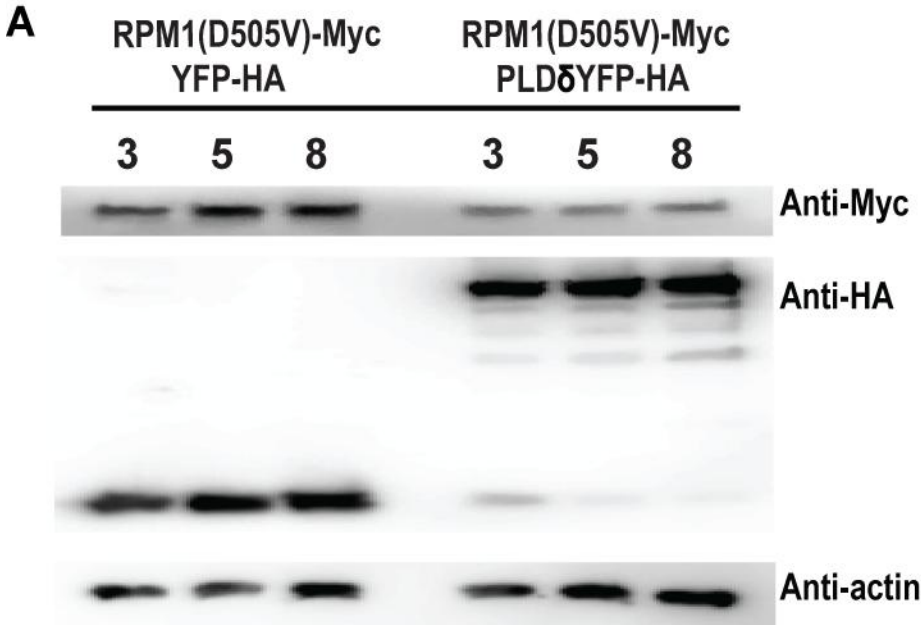

We did not modify the figure. We used a Western Blot System to obtain images. The original files were saved as 16 bit tif version. This kind of file can only be opened with specified Gel document software. We resaved the files with 24 bit tif version. The resaved files are attached.

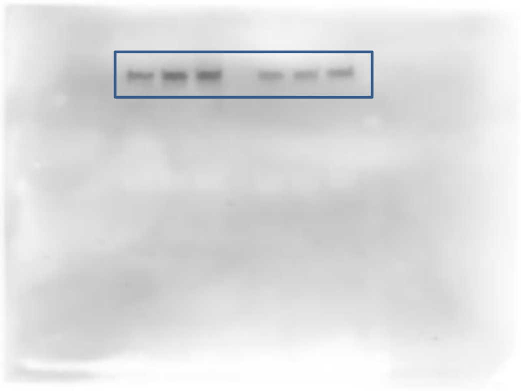

File name: Fig3A-anti-Myc

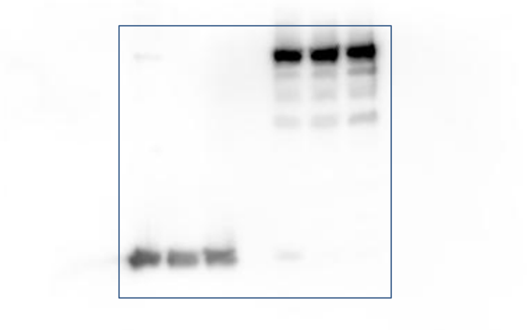

File name: Fig3A-anti-HA

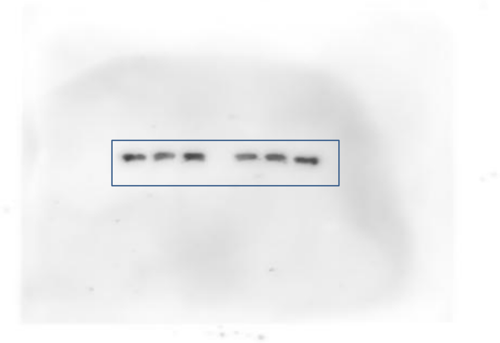

File name: Fig3A-anti-actin

The original files are attached. Selected regions are marked.

Explanation on Figure 3B

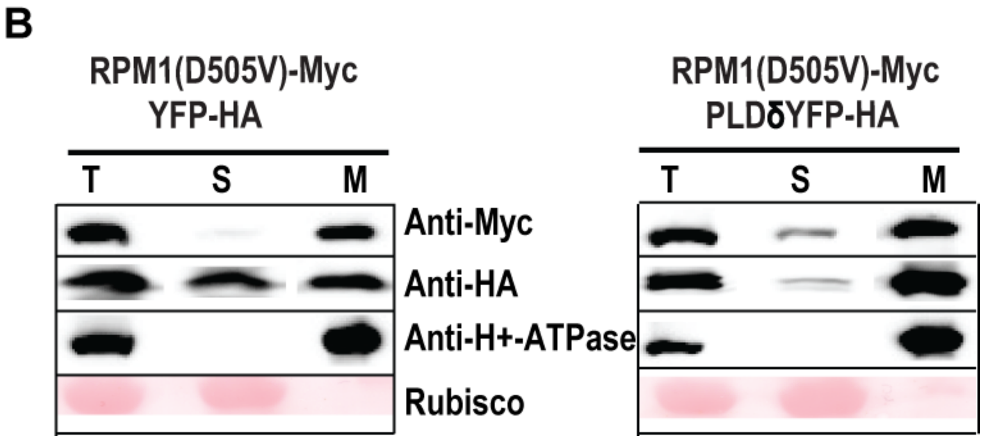

Yes, we modified this figure. The samples were loaded as T, empty well, S , empty well , M. To save space, we cut the empty wells and move the bands together.

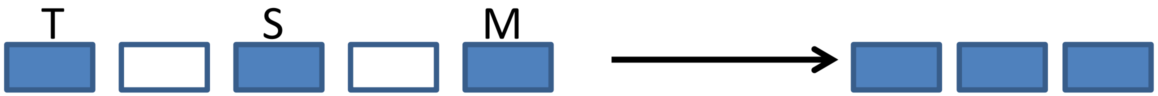

We made a new figure without modification.

Figure without modification

.

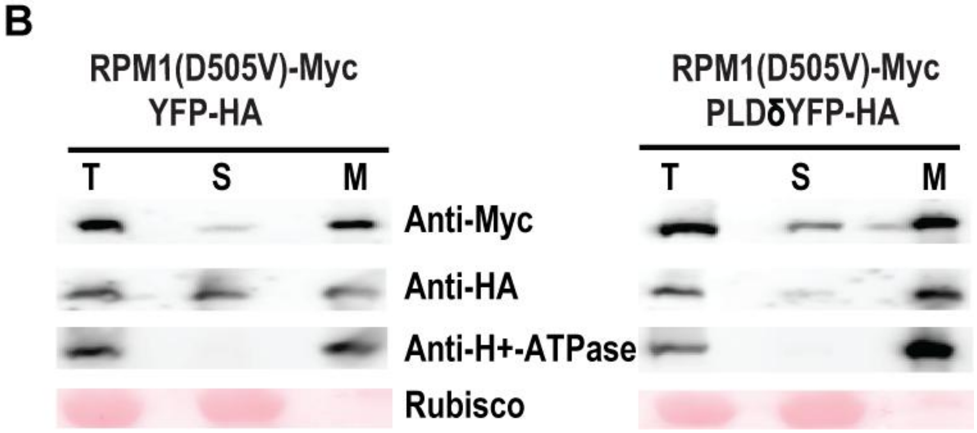

File name: Fig3B-2

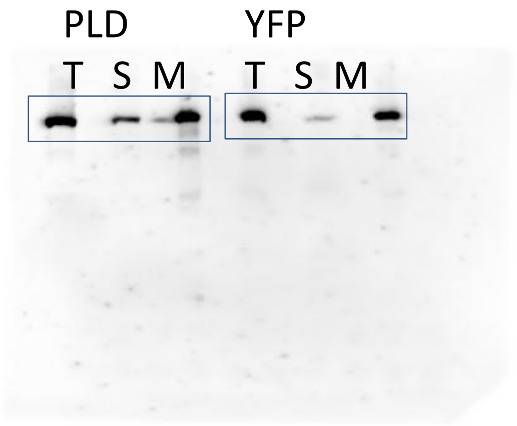

File name: Fig3B-anti-Myc

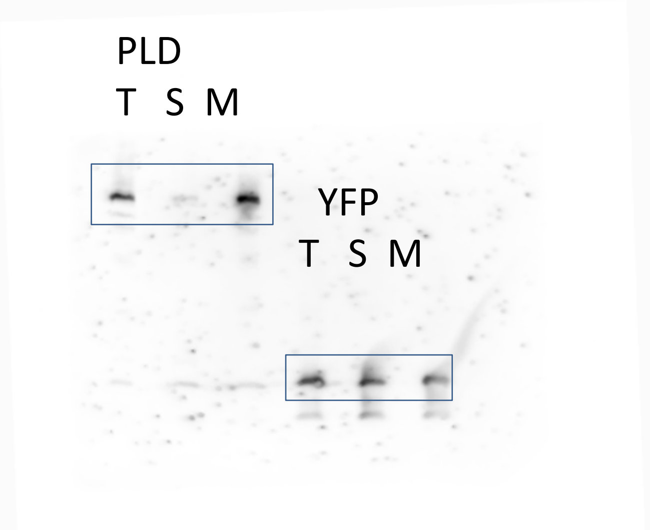

File name: Fig3B-anti-HA

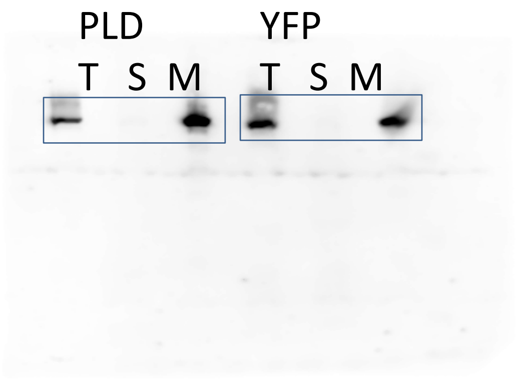

File name: Fig3B-anti-ATPase

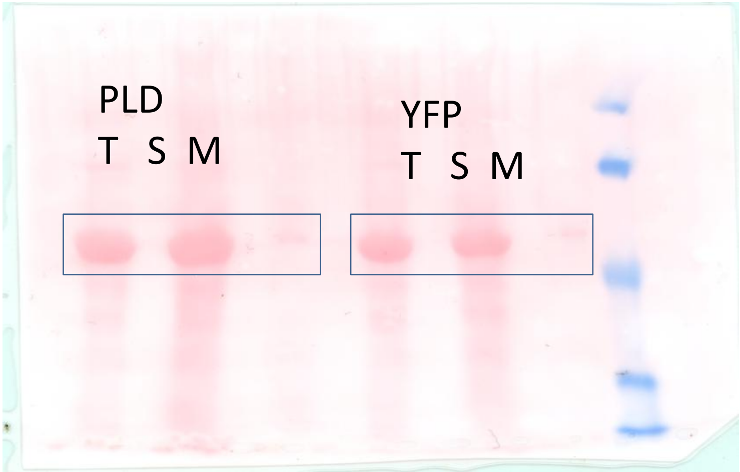

File name: Fig3B-Rubisco

Explanation on Figure 5A

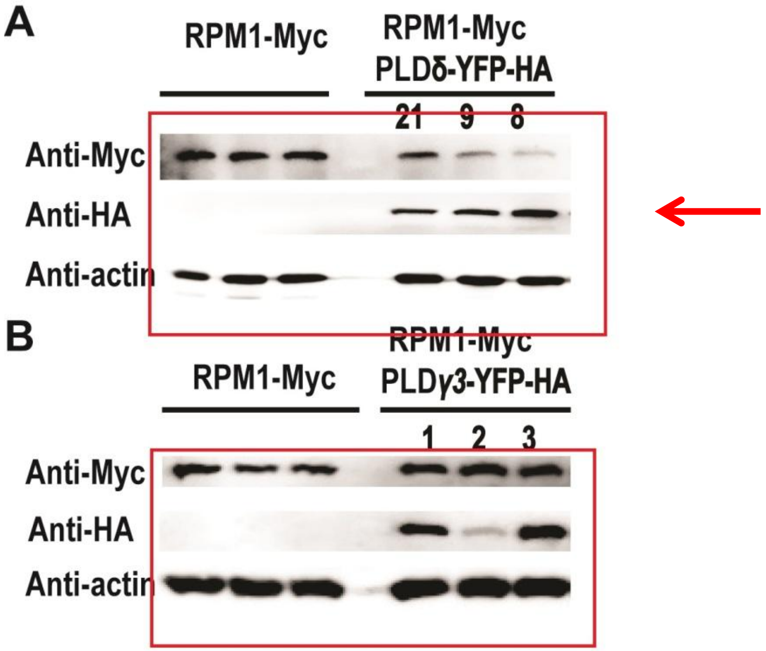

The figure is a cropped image, but no modification. We detected five transgenic lines on the same gel, three representative lines were showed.

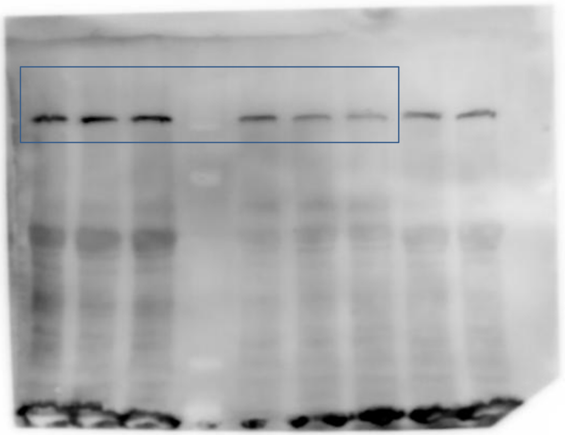

File name: Fig5A-anti-Myc

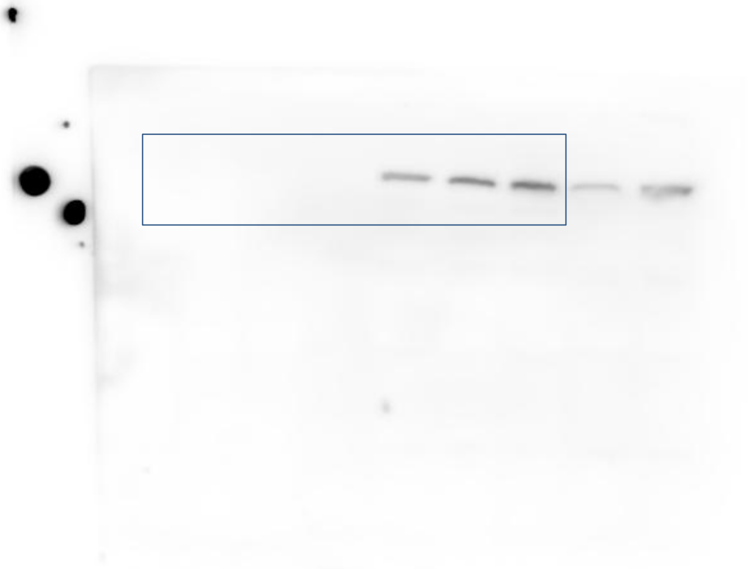

File name: Fig5A-anti-HA

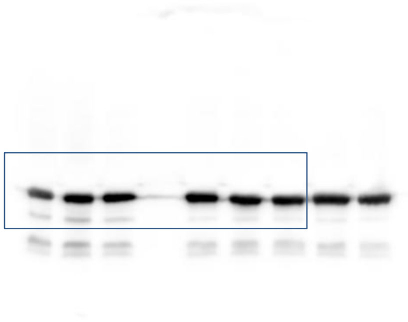

File name: Fig5A-anti-actin

The cropped regions are marked.

Explanation on Figure 5B

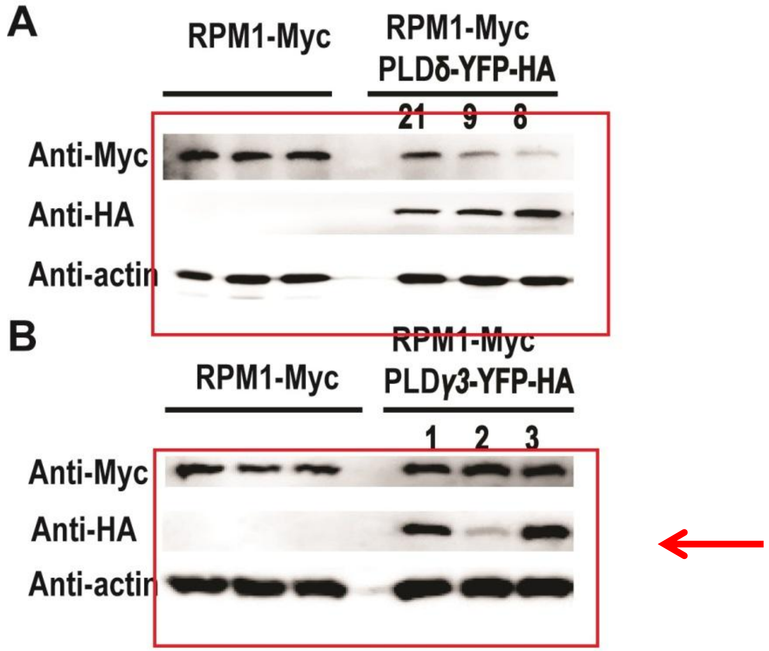

The figure is a cropped image, but no modification. We detected eight transgenic lines on the same gel, three representative lines were showed.

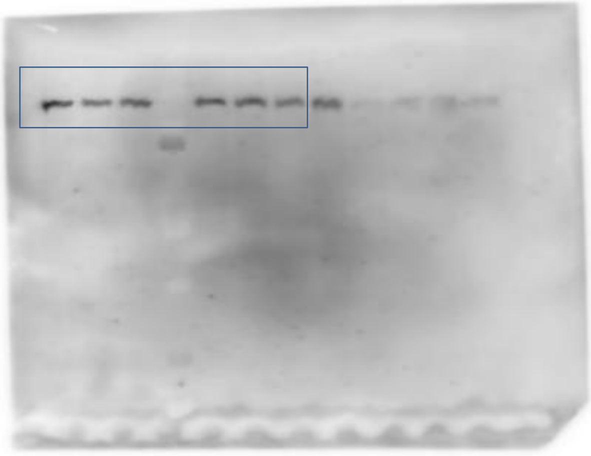

File name: Fig5B-anti-Myc

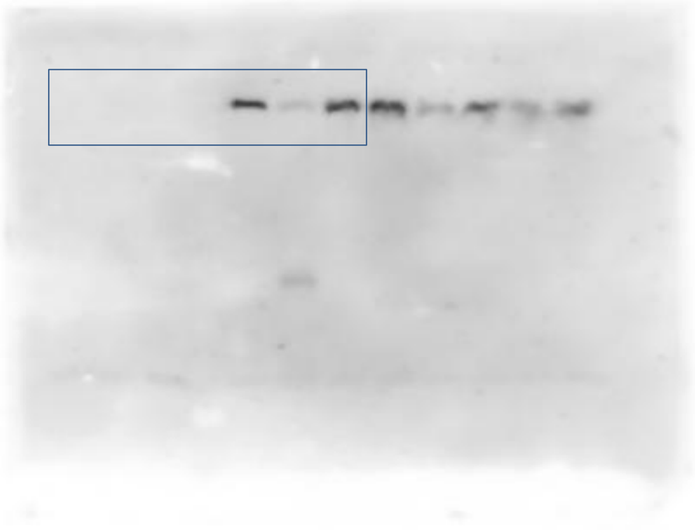

File name: Fig5B-anti-HA

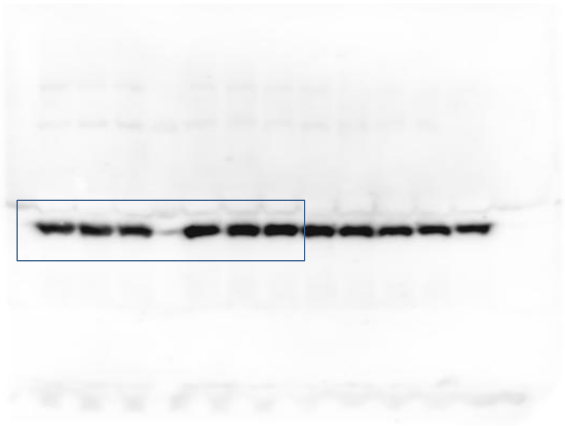

File name: Fig5B-anti-actin

The cropped regions are marked.

Explanation on Figure 7

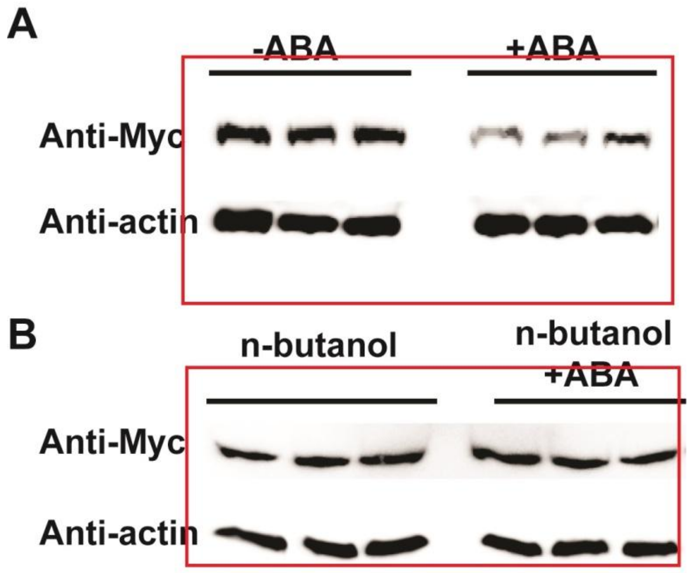

Both Figure A and B are cropped images, but no modification. We detected four ABA-treatment samples on the same gel, three representative samples were showed.

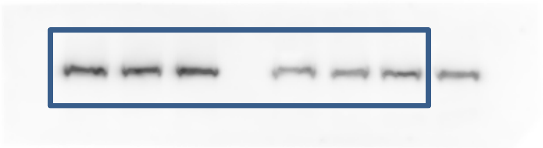

File name: Fig7A-anti-Myc

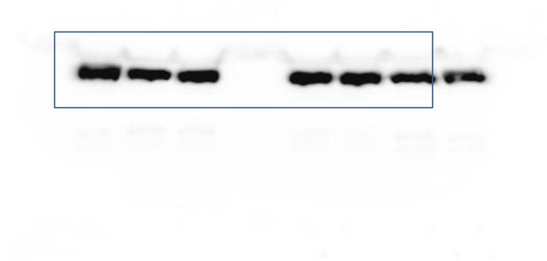

File name: Fig7A-anti-actin

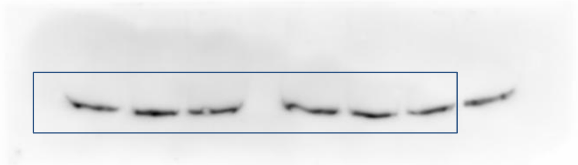

File name: Fig7B-anti-Myc

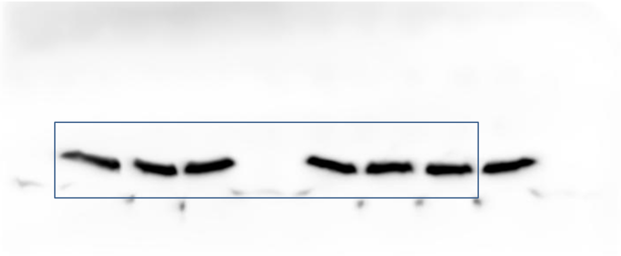

File name: Fig7B-anti-actin

The cropped regions are marked.
